# AGR3 in Breast Cancer: Prognostic Impact and Suitable Serum-Based Biomarker for Early Cancer Detection

**DOI:** 10.1371/journal.pone.0122106

**Published:** 2015-04-15

**Authors:** Stefan Garczyk, Saskia von Stillfried, Wiebke Antonopoulos, Arndt Hartmann, Michael G. Schrauder, Peter A. Fasching, Tobias Anzeneder, Andrea Tannapfel, Yavuz Ergönenc, Ruth Knüchel, Michael Rose, Edgar Dahl

**Affiliations:** 1 Molecular Oncology Group, Institute of Pathology, Medical Faculty of the RWTH Aachen University, Aachen, Germany; 2 Institute of Pathology, University Hospital Erlangen, Erlangen, Germany; 3 Department of Gynaecology and Obstetrics, University Hospital Erlangen, Erlangen, Germany; 4 Patients' Tumor Bank of Hope (PATH) Foundation, München, Germany; 5 Institute of Pathology, Ruhr-University Bochum, Bochum, Germany, on behalf of the PATH Biobank group; 6 Department for Senology, St Anna Hospital, Herne, Germany, on behalf of the PATH Biobank group; The University of Hong Kong, CHINA

## Abstract

Blood-based early detection of breast cancer has recently gained novel momentum, as liquid biopsy diagnostics is a fast emerging field. In this study, we aimed to identify secreted proteins which are up-regulated both in tumour tissue and serum samples of breast cancer patients compared to normal tissue and sera. Based on two independent tissue cohorts (n = 75 and n = 229) and one serum cohort (n = 80) of human breast cancer and healthy serum samples, we characterised AGR3 as a novel potential biomarker both for breast cancer prognosis and early breast cancer detection from blood. AGR3 expression in breast tumours is significantly associated with oestrogen receptor α (P<0.001) and lower tumour grade (P<0.01). Interestingly, AGR3 protein expression correlates with unfavourable outcome in low (G1) and intermediate (G2) grade breast tumours (multivariate hazard ratio: 2.186, 95% CI: 1.008-4.740, P<0.05) indicating an independent prognostic impact. In sera analysed by ELISA technique, AGR3 protein concentration was significantly (P<0.001) elevated in samples from breast cancer patients (n = 40, mainly low stage tumours) compared to healthy controls (n = 40). To develop a suitable biomarker panel for early breast cancer detection, we measured AGR2 protein in human serum samples in parallel. The combined AGR3/AGR2 biomarker panel achieved a sensitivity of 64.5% and a specificity of 89.5% as shown by receiver operating characteristic (ROC) curve statistics. Thus our data clearly show the potential usability of AGR3 and AGR2 as biomarkers for blood-based early detection of human breast cancer.

## Introduction

Breast cancer remains the most frequently diagnosed and leading cause of cancer deaths in women worldwide [1]. Early-stage breast cancer has a favourable prognosis with a 5-year survival rate of up to 90% while this rate declines drastically to 20% upon tumour spreading to distant organs [2]. Therewith, early detection remains a major challenge in the management of breast cancer. Mammography has become the standard of care for breast cancer screening [3] although several limitations are known concerning this procedure, such as a poor accuracy in women with dense breast parenchyma resulting in reduced clinical sensitivity and specificity [3,4]. For women at high risk to develop breast cancer, supplemental magnetic resonance imaging (MRI), an expensive technique that offers excellent imaging even around dense breast tissue, is applied [3]. Unfortunately, the high sensitivity of MRI (85% to 100%) is compromised by a high rate of false positives (37% to 100%) resulting in unnecessary follow-up examinations (including invasive biopsies) causing further stress for the patient and costs [5].

Owing to these limitations of mammography and MRI minimally-invasive novel screening tests are desirable to complement mammography and MRI, or even as stand-alone primary screening tools. Measurement of molecular biomarkers present in bodily fluids (e.g. serum) constitutes a promising tool for the early detection and monitoring of breast cancer. To date, reliable biomarkers for early breast cancer detection and breast cancer monitoring are unavailable or sparse [6]. Determination of serum mucin 1 (MUC-1) and carcinoembryonic antigen (CEA) levels for monitoring of breast cancer patients with metastatic disease during active therapy are the only two circulating biomarkers currently recommended by the American Society of Clinical Oncology (ASCO) as supplementary tests [6]. Thus, finding new circulating biomarkers for breast cancer screening and/or monitoring, but also with prognostic or predictive value, remains an important issue of research.

The human Anterior Gradient (AGR) family consists of three members, namely TXNDC12 (AGR1), AGR2 and AGR3, all belonging to the protein disulfide isomerase (PDI)-related family of proteins [7–9]. AGR2 was first identified in *Xenopus laevis* as the secreted protein XAG-2 involved in differentiation processes [10–12]. Its expression has been shown being up-regulated in various cancers, including pancreas [13], oesophageal [14], lung [15], prostate [16], ovarian [17] and ERα-positive breast cancer [18–21]. Importantly, AGR2 protein concentrations are found to be significantly elevated in serum and/or plasma samples of ovarian [17,22], lung [23] and prostate [24] cancer patients compared to healthy controls proposing AGR2 as a putative cancer serum biomarker in these tumour entities. AGR3, also referred to as breast cancer membrane protein 11 (BCMP11) [25], has been shown being over-expressed in breast [25], ovarian [26] and prostate [27] cancer. Moreover, AGR3 has recently been suggested as a diagnostic marker for intrahepatic cholangiocarcinoma (ICC) [28]. However, the role of AGR3 in carcinogenesis is still obscure. On the one hand, AGR3 was described as a marker of favourable prognosis in serous ovarian cancer [26], whereas a recent publication on the other hand indicated a pro-oncogenic potential for AGR3 demonstrating the mediation of cisplatin resistance by AGR3 in a H1299 cell line xenograft mouse model [29]. In human breast cancer, there have been no studies so far considering the putative biomarker potential of AGR3.

As part of the EU-funded joint research project “MicroBioMed” (Microtechnologies for biomedicine applications), we aimed to identify novel putative protein biomarkers for later integration into a micro-fluidic chip system suitable for early cancer detection or disease monitoring. In the present study, AGR2 and AGR3 were identified for the first time as putative serum protein biomarkers in breast cancer. Moreover, AGR3 was found to be an independent prognostic factor of unfavourable prognosis in lower grade breast cancer cases, indicating a tumour-promoting function in well to moderately differentiated breast carcinomas.

## Materials and Methods

### Cryoconserved clinical specimens

Tumorous and normal breast tissue samples analysed in this study were obtained from the tumour bank of Euregional comprehensive Cancer Center Aachen (ECCA), now part of the RWTH centralized biomaterial bank (RWTH cBMB; http://www.cbmb.rwth-aachen.de). All patients gave written informed consent for retention and analysis of their tissue for research purposes according to local Institutional Review Board (IRB)-approved protocols (approval no. EK-206/09) of the medical faculty at RWTH Aachen University. After surgery, tumour material was immediately snap-frozen in liquid nitrogen. Sections stained with haematoxylin and eosin were prepared for assessing the percentage of tumour and normal epithelial cells, respectively. Only tumour samples containing more than 70% tumour cells, and normal samples containing at least 30% epithelial cells as determined by a pathologist (W.A.), were selected for RNA analysis. Patient characteristics are shown in the supplements (S1 Table).

### Formalin-fixed, paraffin-embedded (FFPE) clinical specimens

AGR3 protein expression was assessed by a pathologist (S.W.) according to an adapted immunoreactive score (IRS) developed by Remmele and Stegner (1987) [30] using a tissue microarray (TMA) described previously [31]. The TMA comprised 190 breast carcinomas and 39 normal breast tissues. Histologically, all tumours were graded according to Bloom and Richardson, as modified by Elston and Ellis [32]. Clinical follow-up data were available for 188 breast cancer patients with a median follow-up period of 138.5 months (range 1–218 months). Clinico-pathologic variables of breast cancer cases included in the tissue microarray are summarised in S2 Table.

### Human serum samples

Human serum samples from breast cancer patients (n = 40) and from cancer-free individuals (n = 40) were obtained from the Patients’ Tumor Bank of Hope (PATH foundation, a research resource for breast cancer biosamples [33]) and the University Hospital of Erlangen. All patients gave written informed consent for retention and analysis of their serum for research purposes according to local Institutional Review Board (IRB)-approved protocols (approval no. EK-255/06 of the medical faculty at Bonn University, EK-3937 and EK-4514 of the medical faculty at Erlangen University). Blood from all patients was drawn immediately or up to 2 days after diagnosis and before starting any cancer-specific treatment. After clotting of blood samples (7.5 ml per patient) obtained by venipuncture, specimens were centrifuged at 1,500 *g* for 10 minutes at room temperature. Isolated serum (3–4 ml) was aliquoted (1.5 ml) and stored at -80°C until use. Clinicopathologic variables of the breast cancer patients are summarised in the supplements (S3 and S4 Tables).

### TCGA patients’ data set

Raw IlluminaHiSeq expression data for *AGR3* as well as the corresponding clinical data of the breast cancer samples analysed (n = 997), were used from The Cancer Genome Atlas (TCGA) [34]. Using sample IDs (see S5 Table), the *AGR3* expression data of breast cancer specimens can be downloaded at the cBio Cancer Genomics Portal (http://www.cbioportal.org) [35], whereas the corresponding clinical data are available at The Cancer Genome Atlas Data Portal (https://tcga-data.nci.nih.gov/tcga/tcgaDownload.jsp).

### RNA extraction and reverse transcription PCR

Total RNA from cryoconserved tissues (20mm^3^ each) was isolated using the standard procedure for TRIzol (Invitrogen, Carlsbad, CA, USA) RNA extraction. Extracted RNA was quantified using the NanoDrop ND1000 spectrophotometer (Thermo Scientific, Waltham, MA, USA). The A260nm/A280nm ratio was generally between 1.9 and 2.0. Subsequently, cDNA was synthesised using 1μg of total RNA and the reverse transcription system (Promega, Madison, WI, USA) according to the manufacturer’s instructions. Briefly, after heat denaturing of RNA (in 8.9μl RNase-free water) for 10min at 70°C, 11.1μl of a mix containing 15U of *AMV* reverse transcriptase, 20U RNase inhibitor and each 0.5μg of both oligo(dT)_15_ and random primers was added to the RNA and the reaction tube was subsequently incubated for 10min at RT, followed by the synthesis step for 15min at 42°C. After cDNA synthesis, enzyme was heat inactivated by incubation for 5min at 95°C. cDNA was stored at -20°C until use.

### Semi-quantitative real-time PCR

cDNAs were amplified by semi-quantitative real-time PCR using SYBR-Green PCR mix (Bio-Rad Laboratories, München, Germany) and the iCycler IQ5 (Bio-Rad Laboratories) as described previously [36]. Gene-specific primer sets for *AGR3* and the reference gene *GAPDH* spanning at least one intron were designed by using Primer3web software (version 4.0.0). Primers for *AGR3* targeting all protein-coding splice variants did not amplify the homologous gene *AGR2*. All reactions were performed in triplicates including negative controls without cDNA. Specificity of amplification products was confirmed by size estimation on agarose gels and melt curve analysis. Obtained data were analysed using the comparative Ct (threshold cycle) method. Complete reaction conditions, primer sequences and lengths of amplicons are listed in S6 Table.

### Western blotting

Each 100ng of recombinant human AGR2 and AGR3 protein (Biorbyt, Cambridge, UK) was diluted 1:2 in 2x NuPAGE LDS Sample Buffer (Invitrogen, Carlsbad, CA, USA) supplemented with 5% dithiothreitol and heat denatured (5min, 95°C). For separation protein was loaded on 4–12% gradient gels (NuPAGE; Invitrogen) and then transferred onto 0.2μm PVDF membranes (Whatman, Dassel, Germany) (1h, 100V) for immunodetection. Blots were blocked in TRIS-buffered saline (TBS) containing 0.1% Tween-20 (TBS-T) and 5% non-fat dry milk (Merck, Darmstadt, Germany) overnight at 4°C. Blocked blots were probed with mouse monoclonal anti-AGR3-antibody (ab82400, Abcam, Cambridge, UK), diluted 1:500 in blocking solution, for 1h at room temperature. After washing (TBS + 0.05% Tween-20), blots were incubated with rabbit anti-mouse (DAKO, Glostrup, Denmark) secondary peroxidase-conjugated antibody, diluted 1:4000 in blocking solution, for 1h at room temperature. After washing (TBS + 0.05% Tween-20), antibody detection was accomplished with Pierce ECL Western blotting Substrate (Thermo Scientific, Rockford, USA). Original uncropped blot is depicted in the supplements (S1 Fig.).

### Immunohistochemistry

Immunohistochemical analysis was carried out according to the manufacturer’s instructions (DAKO 5001; DAKO, Glostrup, Denmark). Heat-induced epitope retrieval was performed in 10mM citrate buffer (pH 6.0) for 10 minutes using a pressure cooker. FFPE sections (3μm) were incubated for 45 minutes with mouse monoclonal anti-AGR3-antibody (1:1000; ab82400, Abcam, Cambridge, UK). AGR3 protein staining was quantified by a pathologist using an adapted immunoreactive scoring system (IRS) according to Remmele and Stegner [30].

### Sandwich Enzyme-Linked Immunosorbent Assay (ELISA)

AGR2 and AGR3 protein serum concentrations were quantitatively assessed by commercially available ELISA kits for AGR2 (E92285Hu, USCN Life Science Inc., Wuhan, China) and AGR3 (Cusabio Biotech, Wuhan, China). Final AGR2 and AGR3 serum concentrations were obtained based on the dilution of samples which corresponded to the linear portion of the standard curve. In order to eliminate plate bias, the location of cancer and control serum samples on the ELISA plate was randomised. Both ELISA assays were performed according to the manufacturer’s instructions.

### Statistical analysis

For statistical analyses SPSS 17.0 (SPSS, Chicago, IL, USA) and GraphPad Prism 5.0 (GraphPad Software, La Jolla, CA, USA) were applied. Differences were considered significant if the two-sided *P* values were < 0.05. To compare two groups the non-parametric Mann-Whitney *U*-test and for comparison of more than two groups the Kruskal-Wallis test was used. The Fisher’s exact test was performed in order to correlate clinico-pathological parameters with AGR3 mRNA or protein expression. Receiver operating characteristic (ROC) curves were calculated to evaluate the diagnostic performance of AGR2, AGR3 and the combination of both. In order to determine the prognostic value of AGR3, univariate Kaplan-Meier survival analysis was performed. To test for an independent prognostic value of AGR3 protein expression, multivariate Cox regression analysis was carried out including only those prognostic factors in the multivariate model that showed statistical significance in univariate log-rank tests.

## Results

### *AGR3* mRNA expression is increased in G1/G2 grade and luminal breast tumours compared to normal tissue controls

**Fig 1:**
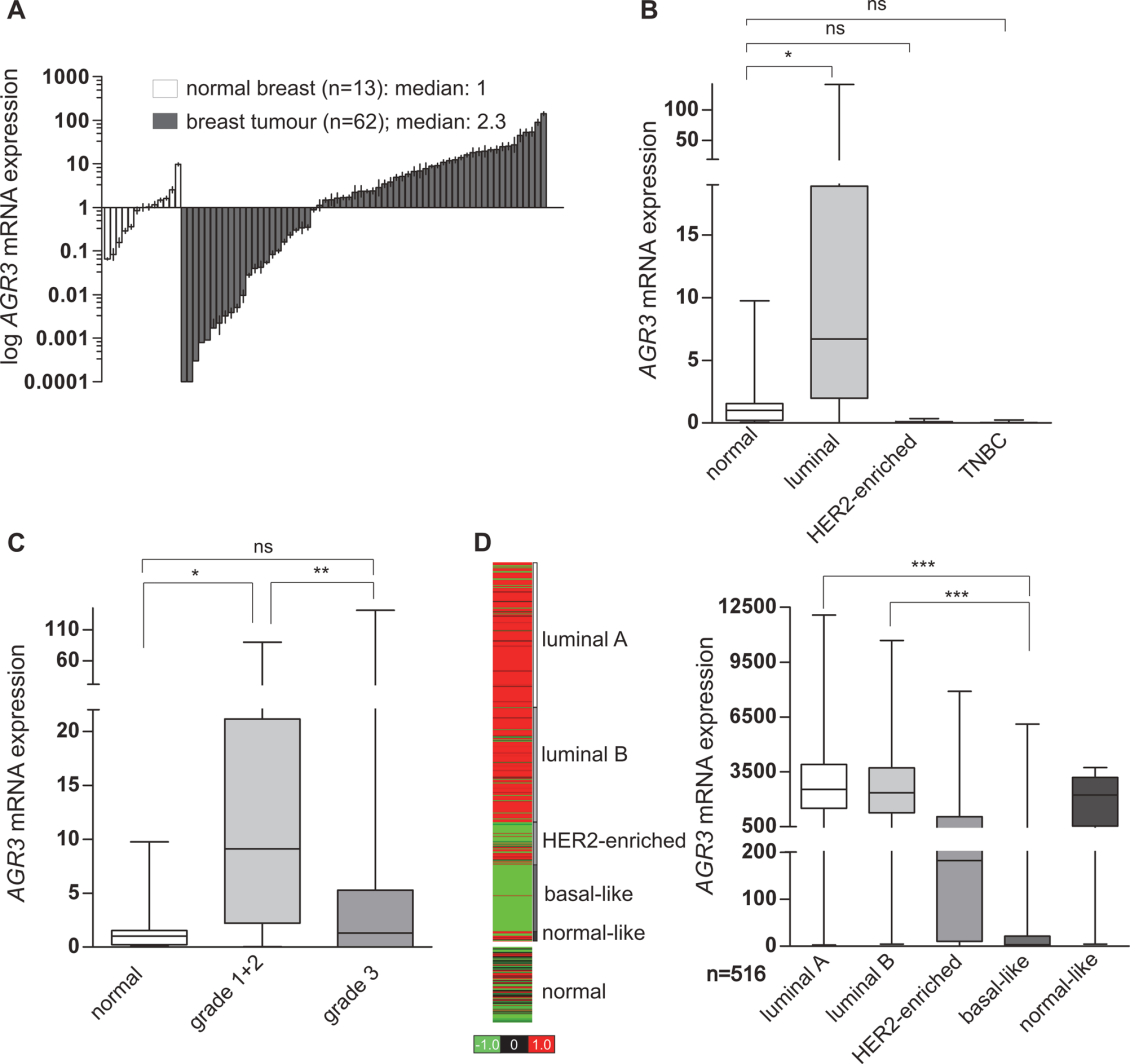
Up-regulation of *AGR3* mRNA expression in G1/G2 grade and luminal breast carcinomas. (**A**) Real-time PCR-based *AGR3* mRNA expression analysis of 62 breast tumour samples compared to 13 healthy breast tissue samples. Vertical lines: ± standard error of margin (SEM). (**B** and **C**) Box plots of the samples shown in A demonstrating a significant association of *AGR3* mRNA expression up-regulation with the IHC-defined luminal breast cancer subtype and lower grading (G1/G2). (**D**) *In silico AGR3* mRNA expression analysis of 516 PAM50-defined breast tumour samples depicted as heat map including normal breast tissues (*left*), and box plot (*right*). Red colour: high, black: intermediate, green: low *AGR3* mRNA expression. Horizontal lines: grouped medians. Boxes: 25–75% quartiles. Vertical lines: range, minimum and maximum. TNBC: triple negative breast cancer; ns: not significant, * *P* < 0.05, ** *P* < 0.01, *** *P* < 0.001.

Though up-regulation of AGR3 expression has recently been shown in breast cancer [21,25], its potential usability as a biomarker in this disease has remained elusive. To assess AGR3 expression data we initially performed a semi-quantitative *AGR3* mRNA expression analysis of 62 breast cancer samples and 13 normal breast tissues. For cohort characteristics of analysed samples see S1 Table. We verified the increased *AGR3* expression in breast tumour samples compared to normal tissues (median fold change (FC): 2.3) (Fig. 1A). Classifying tumour samples by subtypes, i.e. “luminal”, “HER2-enriched” and “triple-negative breast cancer (TNBC)” [37], based on immunohistochemistry (IHC) and fluorescence *in situ* hybridisation (FISH) data for oestrogen receptor (ER), progesterone receptor (PR) and human epidermal growth factor receptor 2 (HER2), revealed a significant (P < 0.05) up-regulation of *AGR3* mRNA expression in luminal breast tumours (Fig. 1B). The association between the luminal subtype and *AGR3* mRNA expression up-regulation was confirmed by Fisher’s exact test that showed a highly significant positive correlation of both a positive ER and PR status with *AGR3* mRNA expression (P < 0.001; S7 Table). In contrast, a reduced expression in HER2-enriched and TNBC tumours was observed compared to normal controls (Fig. 1B). Furthermore, *AGR3* mRNA expression was significantly up-regulated (P < 0.05) in low (G1) and intermediate (G2) grade tumours compared to normal breast tissues, but not in high grade (G3) breast cancer cases (Fig. 1C). This association between *AGR3* mRNA expression and the tumour grades G1 and G2 was further corroborated performing Fisher’s exact test (P < 0.01; S7 Table).

To evaluate the significance of our data, we analysed *AGR3* expression in a large dataset of an independent study. Using data of 516 breast cancer patients available at *The Cancer Genome Atlas* (TCGA) [34] we found pronounced *AGR3* mRNA expression in both PAM50 [38] luminal subtypes, i.e. luminal A and B tumours, with a slightly stronger expression in luminal A carcinomas (Fig. 1D). In turn, a significantly lower expression (median FC compared to luminal subtype: 770) was observed in PAM50 basal-like breast tumours (for both P < 0.001), thus confirming the results obtained in our data set (Fig. 1D).

### AGR3 protein over-expression in human breast tumours

**Fig 2:**
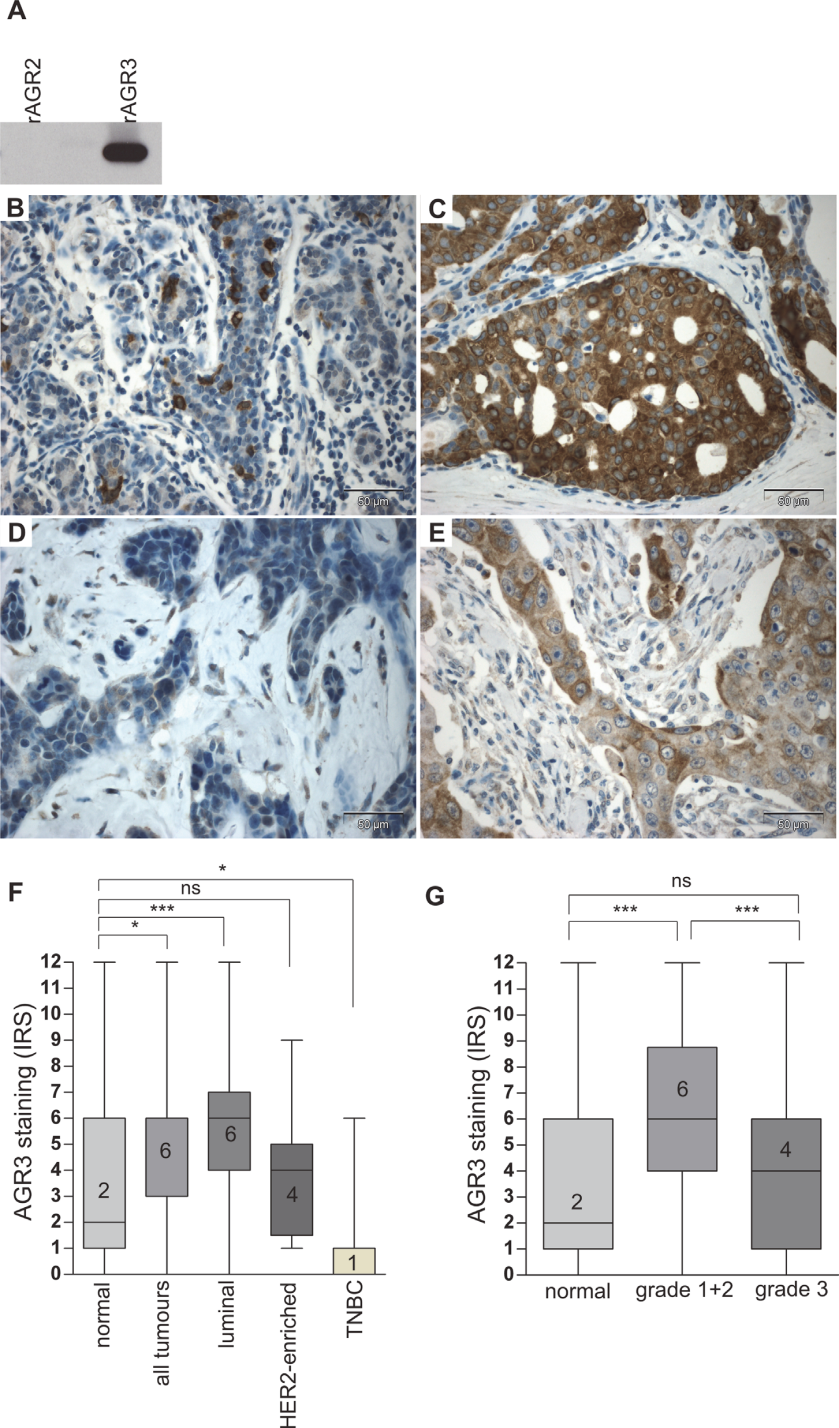
Up-regulation of AGR3 protein in G1/G2 grade and luminal breast cancer. **(A)** Western blot detection of human recombinant AGR3 (rAGR3, 100ng) but not recombinant AGR2 (rAGR2, 100ng) by monoclonal AGR3 antibody used for IHC analysis. **(B)** Cytoplasmic staining of AGR3 in isolated cells of the normal breast epithelium. **(C)** Strong cytoplasmic AGR3 protein expression in epithelial cancer cells of an IHC-defined luminal breast tumour. **(D)** Absent and **(E)** weak cytoplasmic AGR3 expression in two different triple negative breast tumours. **(F)** Box plot analysis demonstrating a significant up-regulation of AGR3 in all tumours (n = 190) and the luminal subtype (n = 113), but a significant reduction of expression in the triple negative breast cancer cases (TNBC, n = 23) compared to normal breast tissues (n = 39). **(G)** Box plot analysis showing a highly significant up-regulation of AGR3 in G1 and G2 breast tumours (n = 104) compared to normal controls (n = 39). Horizontal lines: grouped medians. Boxes: 25–75% quartiles. Vertical lines: range, minimum and maximum. Ns: not significant, * *P* < 0.05, *** *P* < 0.001. IRS: immunoreactive score.

Next, we analysed AGR3 expression on protein level. We conducted an immunohistochemical analysis for AGR3 using a tissue microarray consisting of 39 normal breast tissues and 190 breast tumours (see S2 Table for cohort characteristics). To verify the antibody’s specificity for AGR3 and to exclude cross-reactivity with the homologous protein AGR2, we performed a Western blot experiment using recombinant AGR2 and AGR3 protein. The antibody showed excellent specificity for AGR3 and no cross-reactivity with AGR2 (Fig. 2A). AGR3 protein staining was quantified according to an adapted immunoreactive score (IRS) developed by Remmele and Stegner (1987) [30]. AGR3 was predominantly expressed in the cytoplasm of breast tumour cells whereas only sporadic expression in single cells of the healthy breast epithelium was noticed (Fig. 2B-E). In concordance with our *AGR3* mRNA data abundant AGR3 protein expression was found in tumours of luminal subtype (Fig. 2C) whereas only weak or absent AGR3 protein staining was observed in TNBC tumours (Fig. 2D and E). Statistically quantifying AGR3 protein expression we observed a significant over-expression (P < 0.05) of AGR3 in tumour samples compared with normal breast tissues (Fig. 2F). AGR3 protein expression levels of both luminal and TNBC cases were significantly different (P < 0.001 and P < 0.05) from that determined in normal breast tissues, again being in line with our *AGR3* mRNA expression data. No significant differential AGR3 protein expression was observed between normal breast tissues and the HER2-enriched breast carcinomas. Furthermore, a highly significant (P < 0.001) over-expression of AGR3 protein in G1 and G2 breast tumours compared to normal breast tissues (Fig. 2G) was detectable as well. The correlation between AGR3 protein expression with both, the luminal subtype and the histological grades G1 and G2 was finally supported by Fisher’s exact test (S8 Table).

### AGR3 protein expression predicts unfavourable tumour-specific survival in low and intermediate grade tumours

**Fig 3:**
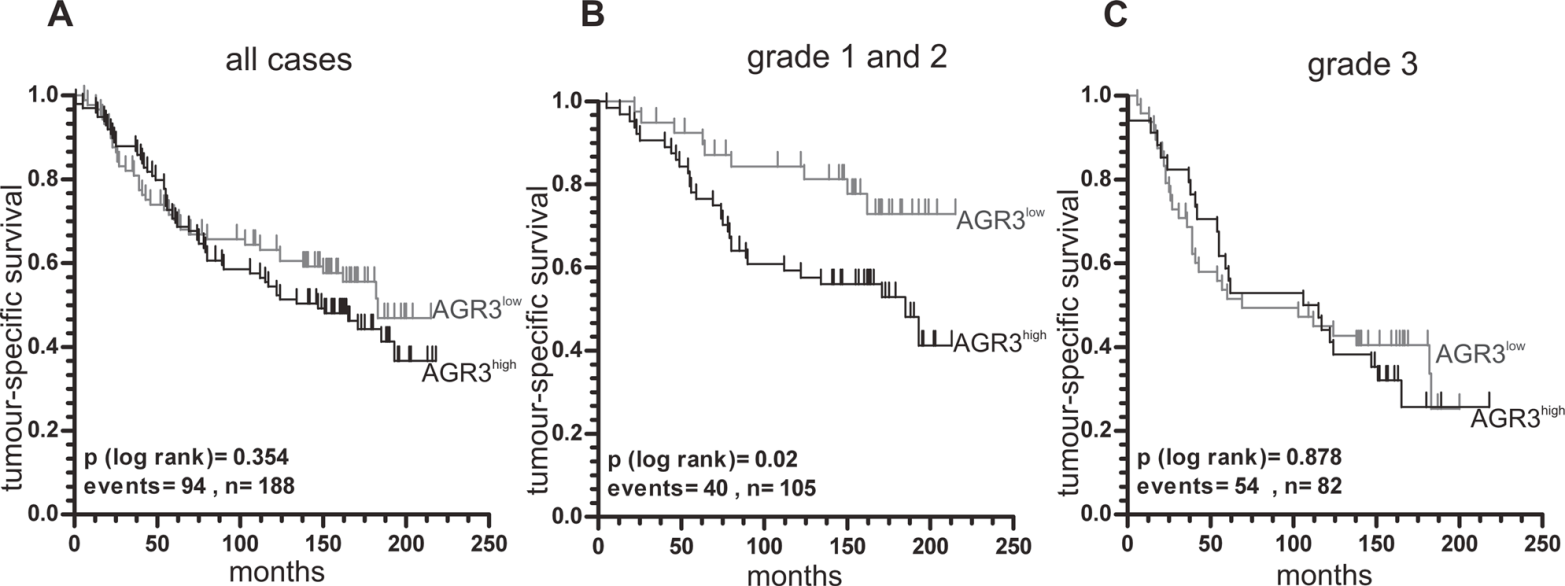
Increased AGR3 protein expression is associated with reduced tumour-specific survival in G1 and G2 breast cancer patients. According to the median AGR3 IRS of 6, the two groups “low AGR3” (IRS = 0–4) and “high AGR3” (IRS = 6–12) were dichotomised. Univariate Kaplan-Meier survival curves displaying tumour-specific survival of patients with low AGR3 expression (grey line) in relation to high AGR3 expression (black line) in (**A**) all, (**B**) low and intermediate (G1 and G2) and (**C**) high-grade (G3) breast cancer cases.

So far, the prognostic impact of AGR3 has not yet been investigated in breast cancer. Therefore, we analysed the impact of AGR3 protein expression on patients’ outcome in 190 breast cancer samples (see S2 Table). Considering all tumours, there was no significant association between AGR3 expression and patient tumour-specific survival (Fig. 3A). After subgrouping our cohort, we found that AGR3 has a significant (P < 0.05) prognostic impact in the low (G1) and intermediate (G2) grade tumours (Fig. 3B and Table 1), but not in the group of high grade (G3) cases (Fig. 3C). Patients with low and intermediate grade tumours showing high AGR3 expression had an unfavourable outcome (mean tumour-specific survival: 142.5 months ± 9.6; 95% CI: 123.8–161.2) compared to those with low AGR3 expression (mean tumour-specific survival: 181.7 months ± 10.1; 95% CI: 162.0–201.4). To validate whether the significance of the relationship between AGR3 expression and patient survival is independent of other tumour variables, we performed Cox’s multivariate analysis including only those variables that showed significance in univariate analysis (Table 2). The Cox regression model confirmed AGR3 to be a putative independent marker of unfavourable prognosis in low and intermediate grade breast tumours (multivariate hazard ratio: 2.186, 95% CI: 1.008–4.740, P < 0.05).

**Table 1:** Univariate analysis of clinico-pathological parameters influencing tumour-specific survival in the group of grade^a^ 1 and 2 breast tumours.

**Parameter**	**Tumour-specific survival**		
** **	**n**	**events**	**P-value** ^b^
AGR3 protein expression^c^			
AGR3^low^	40	9	
AGR3^high^	64	31	**0.02**
Age at diagnosis			
<54,5 years	52	13	
≥54,5 years	52	27	**0.002**
Tumour size^d^			
pT1	44	8	
pT2-4	59	31	**<0.001**
Lymph node status^d^			
pN0	53	14	
pN1-3	47	22	**0.016**
Histological type			
invasive ductal	92	35	
invasive lobular	5	3	0.165
Oestrogen receptor status			
negative (IRS^e^ 0–2)	15	6	
positive (IRS^e^ 3–12)	59	21	0.636
Progesterone receptor status			
negative (IRS^e^ 0–2)	45	20	
positive (IRS^e^ 3–12)	39	12	0.108
HER2 status^f^			
negative (0; 1+; 2+)	75	31	
positive (3+)	12	2	0.270

^a^According to Bloom and Richardson, as modified by Elston and Ellis [32].

^b^Log-rank test at the two-sided significance level of 0.05.

^c^Median immunoreactive score (IRS) according to Remmele and Stegner [30] was used as cut-off: AGR3 low (IRS 0–4), AGR3 high (IRS 6–12).

^d^According to TNM classification by Sobin and Wittekind [58].

^e^Immunoreactive score (IRS) according to Remmele and Stegner [30].

^f^Overexpression of the *ERBB2* gene (Her-2/neu) was diagnosed analogously to the threshold of the DAKO-Score system based on IHC assay. Significant P-values are marked in bold face.

**Table 2:** Multivariate Cox regression analysis including all factors potentially influencing tumour-specific survival in grade^a^ 1 and 2 breast cancer samples.

**Variable **	**HR**	**P-value**	**95%CI**	
			**lower**	**upper**
AGR3 protein expression^b^				
AGR3^low^	1.000			
AGR3^high^	2.186	**0.048**	1.008	4.740
Tumour size^c^				
pT1	1.000			
pT2-4	3.453	**0.005**	1.455	8.194
Lymph node status^c^				
pN0	1.000			
pN1-3	1.879	0.087	0.912	3.869
Age at diagnosis				
<54.5 years	1.000			
≥54.5 years	2.481	**0.011**	1.231	4.999

^a^According to Bloom and Richardson, as modified by Elston and Ellis [32].

^b^Median immunoreactive score (IRS) according to Remmele and Stegner [30] was used as cut-off: AGR3 low (IRS 0–4), AGR3 high (IRS 6–12).

^c^According to TNM classification by Sobin and Wittekind [58]. Significant P-values are marked in bold face. HR, hazard ratio; CI, confidence interval.

### Analysing AGR3 and AGR2 as potential blood-based biomarkers for breast cancer detection

**Fig 4:**
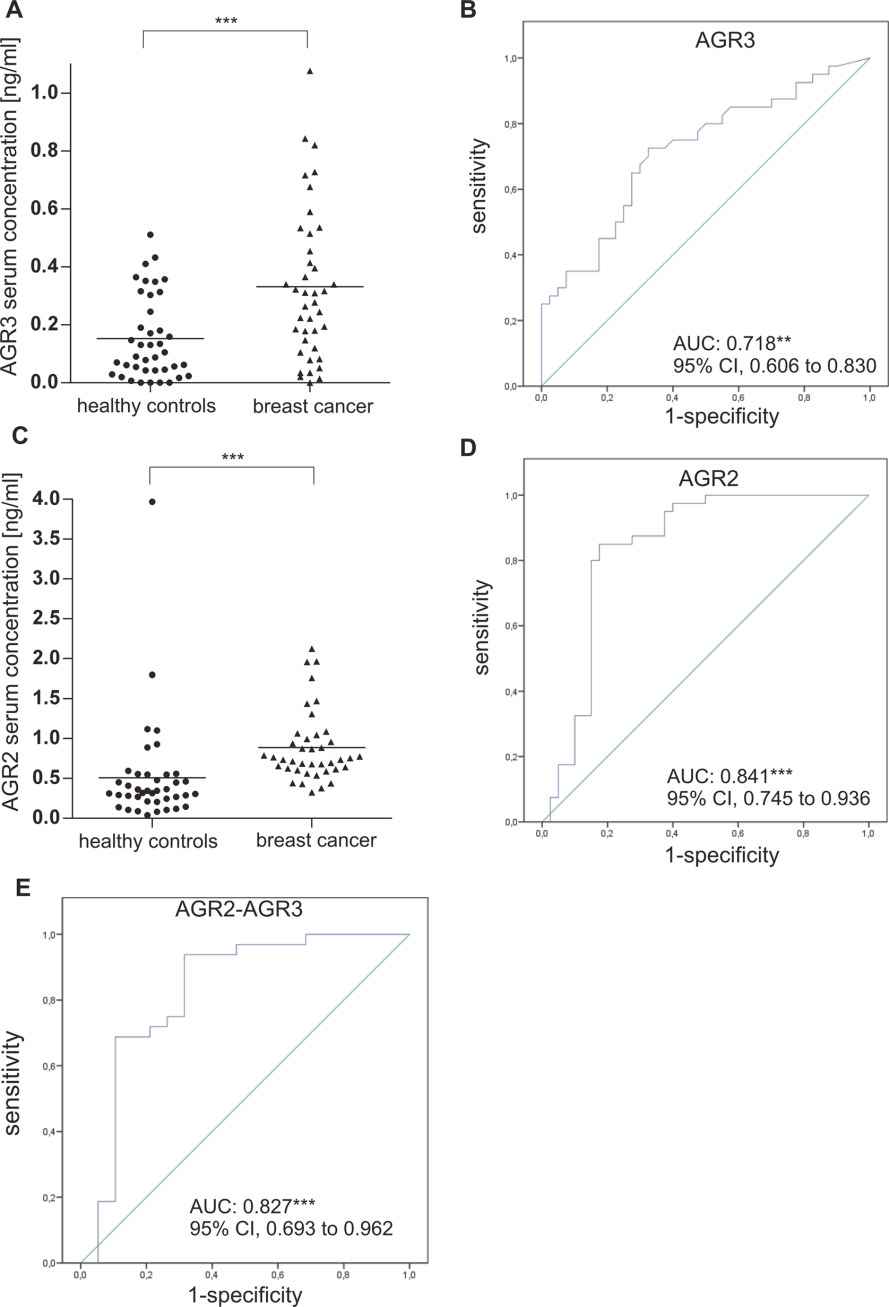
Evaluation of the diagnostic performance of candidate serum biomarkers AGR3, AGR2 and their combination. Scatter plot demonstrating a highly significant elevation of (**A**) AGR3 and (**C**) AGR2 protein concentration in human serum samples from breast cancer patients (n = 40) in comparison with samples from healthy women (n = 40). ROC curve analysis for (**B**) AGR3, (**D**) AGR2 and (**E**) the combination of both proteins. ** *P* < 0.01, *** *P* < 0.001. ROC: receiver operating characteristic, AUC: area under the curve, 95% CI: 95% confidence interval.

In light of the given over-expression of AGR3 in breast tumours associated with a potential prognostic impact, we subsequently assessed whether AGR3 protein concentration is increased in human serum samples of breast cancer patients as well. We determined the AGR3 protein level in an age-matched cohort of 40 serum pairs (i.e. 40 serum samples derived from breast cancer patients and 40 sera derived from healthy donors) using a commercially available ELISA kit. The majority of cancer sera was obtained from patients with low stage breast tumours (see S3 Table for cohort characteristics). Indeed, we demonstrated a significantly increased AGR3 serum concentration (P < 0.001, median FC: 3.0) in breast cancer serum samples in comparison to control samples from healthy donors (Fig. 4A). By performing receiver operating characteristic (ROC) statistics we further aimed to determine the clinical performance of AGR3 as a putative biomarker for breast cancer detection. Based on AGR3 protein level in serum samples, we were able to discriminate between breast cancer patients and healthy women with a sensitivity of 35% and a specificity of 92.5% (P < 0.01; AUC: 0.718 (95% CI, 0.606 to 0.830)) (Fig. 4B).

In order to increase the clinical sensitivity we focused on a further putative biomarker candidate, namely AGR2, that is the probable paralogue of AGR3 [21]. Meanwhile, AGR2 has been described as a serum biomarker for ovarian [17], lung [23] and prostate cancer [24]. Despite its strong up-regulation in breast cancer and probable secretion [20], its potential as a serum protein biomarker for breast cancer has not been investigated so far. Using a well-established ELISA assay [24,39], we measured the AGR2 concentration in an age-matched cohort of 40 serum pairs, slightly differing in composition from that used for AGR3 screening (S4 Table). We revealed a highly significant elevation (P < 0.001, median FC: 2.2) of AGR2 protein level in breast cancer sera compared to controls (Fig. 4C). Next, we performed receiver operating characteristic (ROC) curve analysis for AGR2 and the combination of both proteins, i.e. AGR2 and AGR3 as a biomarker duplet. Based on AGR2, a sensitivity of 32.5% and a specificity of 90% was achieved (P < 0.001; AUC, 0.841 (95% CI, 0.745 to 0.936)) (Fig. 4D). Importantly, a combined AGR3 and AGR2 biomarker evaluation led to an increased sensitivity value of 64.5%, maintaining in parallel a high specificity of 89.5% (AUC, 0.827 (95% CI, 0.693 to 0.962)) (Fig. 4E).

## Discussion

The multidisciplinary EU-funded MicroBioMed project aims at developing a cost-effective, sensitive and specific liquid biopsy based chip system with possible applications in the complementation of screening mammography and disease monitoring. In the course of this study we aimed to identify novel serum protein biomarker candidates with sufficient clinical specificity and sensitivity that can be integrated into a lab-on-chip system.

Today, several lines of evidence suggest a potential role for AGR3, a member of the protein disulfide isomerase (PDI)-related family of proteins [8], in breast carcinogenesis. AGR3 has recently been described as up-regulated in breast cancer compared to normal breast tissues [25], while the putative clinical impact of AGR3 over-expression in breast cancer as prognostic or diagnostic protein biomarker remains elusive. The current study is the first to analyse in depth the AGR3 expression, as well as its potential clinical relevance in breast cancer. Initially, we demonstrated a clear up-regulation of AGR3 expression in human breast carcinomas compared to normal breast tissues on both mRNA and protein level. Interestingly, concerning the distinct breast cancer subtypes, AGR3 expression was significantly higher in luminal breast cancer compared with the triple negative breast cancer cases. Moreover, a correlation of AGR3 mRNA and protein expression with low and intermediate (G1 and G2) grade breast tumours was identified. These data are in concordance with a recent study, proposing a positive correlation of AGR3 with oestrogen receptor expression and lower tumour grade in a smaller breast cancer sample collection [21].

This observation was furthermore underscored by analysing AGR3 mRNA expression data of breast cancer samples of the TCGA platform [34]. In line, TCGA data analyses revealed an increased *AGR3* mRNA expression in luminal breast cancer compared with basal-like tumours.

Moreover, we examined for the first time the putative prognostic relevance of AGR3 in breast cancer. Univariate analysis demonstrated that patients with low and intermediate grade tumours showing high AGR3 protein expression had a significantly reduced tumour-specific survival compared to those with low AGR3 expression. The Cox regression model confirmed AGR3 to be a putative independent marker of unfavourable prognosis in G1 and G2 breast tumours. So far, the only study having considered the putative prognostic value of AGR3 reported the protein to be a biomarker of favourable prognosis in serous ovarian cancer [26]. However, a recent study by Gray *et al*. demonstrating the mediation of cisplatin resistance by AGR3 over-expression in an H1299 cell line xenograft mouse model indicates tumour-promoting properties of AGR3 [29]. Furthermore, our results are in line with recent findings for the homologous protein AGR2 that is now discussed as being a prognostic factor of an adverse prognosis in breast cancer [40,41]. In contrast to the well described pro-oncogenic factor AGR2 [42], functional studies for AGR3 are missing. Interestingly, AGR2 and AGR3 have both been shown to interact with the protein C4.4A (LYPD3) [21], a factor implicated in tumour progression [43]. In conclusion, our data propose a pro-oncogenic impact of AGR3 in breast cancer, at least in well to moderately differentiated breast carcinomas.

Beside the potential prognostic relevance of AGR3 in G1 and G2 grade breast tumours, AGR3 is also an attractive serum based biomarker candidate. Using a commercially available ELISA we demonstrated for the first time a significantly increased AGR3 protein serum concentration in sera from breast cancer patients compared to samples from age-matched healthy individuals. It is noteworthy that the majority of the analysed cancer sera were from low stage breast cancer patients suggesting a potential utility of AGR3 for early detection of breast cancer. To evaluate the diagnostic performance of AGR3 we performed ROC curve analysis resulting in a high specificity of 92.5% and a sensitivity of 35%.

It is obvious that single protein biomarkers are not reliable to detect cancer with high accuracy. Instead, panels of biomarkers will be necessary to ensure early detection and monitoring of malignant diseases with both high specificity and sensitivity [44]. Thus, we aimed to identify a further novel biomarker candidate in breast cancer and focused on AGR2, the probable paralogue of AGR3 previously described as up-regulated in breast cancer [19,20]. As already reported for other ER-resident proteins in cancer [45], AGR2 protein concentration has also been found increased in serum or plasma samples of ovarian, lung and prostate cancer patients compared to healthy controls [17,22–24]. However, thus far the potential utility of AGR2 protein as a serum biomarker for breast cancer has not yet been investigated. Using a well-established commercial ELISA for AGR2 [24,39], we detected significantly elevated AGR2 protein levels in breast cancer sera in comparison to healthy controls. ROC curve analysis for AGR2 revealed similar values for specificity (90%) and sensitivity (32.5%) as shown for AGR3. Importantly, the combined AGR3 and AGR2 performance led to an increased sensitivity value of 64.5%, maintaining in parallel a high specificity of 89.5%.

To date no recommendation was pronounced for any biomarker-based early breast cancer screening test. So far, early diagnosis of breast cancer mainly relies on screening mammography although its limitations especially in women with dense breast tissue are well-recognised [3,46]. Inconclusive imaging often leads to further follow-up examinations, including invasive biopsies, ending in additional distress for the patient and immense costs [47–49]. A combined integration of the here identified putative serum biomarkers AGR2 and AGR3 into a microfluidic-based analysing platform may help to improve breast cancer detection, hence to discriminate between healthy and disease status. These point-of-care biosensor systems have emerged as powerful tools for the diagnosis and monitoring of cancer and hold the promise to fulfil high-throughput and high-precision screening with reduced costs [44]. Not unexpectedly, there is a growing number of studies aiming at developing novel immunoassay-based biosensor platforms to detect cancer [50,51] contributing to novel approaches in the emerging field of liquid biopsy. However, the clinical performance of such microfluidic systems closely depends on used biomarkers which finally caused the limitations of the usability for early detection or monitoring of cancer. Mostly, microfluidic systems have been applied to the isolation and detection of circulating tumour cells (CTCs) that may be especially helpful in monitoring of disease recurrence and management of therapeutic strategies for patients with metastasised malignancies using whole genome techniques such as Next Generation Sequencing (NGS) to predict outcome or therapy response [52]. However, the impact of CTCs for early cancer detection is controversial. Although there are hints that CTCs can be found in the circulation of patients with low-stage disease [53], the incidence and count of CTCs seem to strongly depend on tumour stage and metastasis [54]. Moreover, the heterogeneity of epithelial surface markers, necessary to specifically isolate CTCs from blood, is complex and could be hampered by processes such as the epithelial-to-mesenchymal transition (EMT) [55]. To avoid false-positive results due to contamination with normal blood or epithelial cells, phenotyping of isolated cells by methods like immunostaining or genomic analysis is necessary. Furthermore, owing to low CTC counts in the circulation of patients with low-stage disease and limited sensitivity of current chip-devices, large volumes of blood samples are required [52]. In contrast to that, molecular biomarkers such as free-circulating DNA [56] or soluble proteins [57] encoded by putative oncogenes have a great potential to detect early disease stages and both the sensitivity and specificity of those biomarkers can be improved by developing a panel of single-biomarkers usable for early cancer detection [56]. Owing to that, further validation steps using independent serum collections have to be addressed in future studies to strengthen the robustness of our identified AGR2/AGR3 biomarker duplet performance potentially completed by further biomarker candidates increasing the clinical sensitivity in the finally developed chip system.

In conclusion, our study suggests a putative prognostic impact of AGR3 in low (G1) and intermediate (G2) grade breast carcinomas. Moreover, we demonstrate that both proteins AGR3 and AGR2 are detectable by ELISA technique at significantly elevated concentrations in sera from breast cancer patients compared with age-matched serum samples from healthy women. Hence, our findings clearly show for the first time the potential usability of AGR3 and AGR2 as biomarkers for non-invasive early detection of human breast cancer.

## Supporting Information

S1 Table Cohort characteristics of cryoconserved breast cancer tissue specimens. (DOC)

S2 Table Cohort characteristics of FFPE breast cancer tissue specimens. (DOC)

S3 Table Cohort characteristics of breast cancer serum specimens analysed for AGR3 protein concentration. (DOC)

S4 Table Cohort characteristics of breast cancer serum specimens analysed for AGR2 protein concentration. (DOC)

S5 Table TCGA breast cancer sample IDs. (XLS)

S6 Table Primer sequences and rt-qPCR conditions for *AGR3* mRNA expression analysis. (DOC)

S7 Table Clinico-pathological parameters of cryoconserved breast cancer tissue specimens in relation to *AGR3* mRNA expression. (DOC)

S8 Table Clinico-pathological parameters of FFPE breast cancer tissue specimens in relation to AGR3 protein expression. (DOC)

S1 Fig Original uncropped Western blot: antibody specificity control for AGR3. (PPT)

## References

[1] Jemal A, Bray F, Center MM, Ferlay J, Ward E, Forman D. Global cancer statistics. CA Cancer J Clin. 2011;61: 69–90. 10.3322/caac.20107 21296855

[2] Etzioni R, Urban N, Ramsey S, McIntosh M, Schwartz S, Reid B, et al The case for early detection. Nat Rev Cancer. 2003;3: 243–52. 12671663 10.1038/nrc1041

[3] Berg WA. Tailored supplemental screening for breast cancer: what now and what next? AJR Am J Roentgenol. 2009;192: 390–9. 10.2214/AJR.08.1706 19155400

[4] Armstrong K, Moye E, Williams S, Berlin JA, Reynolds EE. Screening mammography in women 40 to 49 years of age: a systematic review for the American College of Physicians. Ann Intern Med. 2007;146: 516–26. 17404354 10.7326/0003-4819-146-7-200704030-00008

[5] Hooley RJ, Andrejeva L, Scoutt LM. Breast cancer screening and problem solving using mammography, ultrasound, and magnetic resonance imaging. Ultrasound Q. 2011;27: 23–47. 10.1097/RUQ.0b013e31820e15ac 21343800

[6] Harris L, Fritsche H, Mennel R, Norton L, Ravdin P, Taube S, et al American Society of Clinical Oncology 2007 update of recommendations for the use of tumor markers in breast cancer. J Clin Oncol. 2007;25: 5287–312. 17954709 10.1200/JCO.2007.14.2364

[7] Alanen HI, Williamson RA, Howard MJ, Lappi A-K, Jäntti HP, Rautio SM, et al Functional characterization of ERp18, a new endoplasmic reticulum-located thioredoxin superfamily member. J Biol Chem. 2003;278: 28912–20. 12761212 10.1074/jbc.M304598200

[8] Persson S, Rosenquist M, Knoblach B, Khosravi-Far R, Sommarin M, Michalak M. Diversity of the protein disulfide isomerase family: identification of breast tumor induced Hag2 and Hag3 as novel members of the protein family. Mol Phylogenet Evol. 2005;36: 734–40. 15935701 10.1016/j.ympev.2005.04.002

[9] Ivanova AS, Tereshina MB, Ermakova G V, Belousov V V, Zaraisky AG. Agr genes, missing in amniotes, are involved in the body appendages regeneration in frog tadpoles. Sci Rep. 2013;3: 1279 10.1038/srep01279 23412115 PMC3573343

[10] Bradley L, Wainstock D, Sive H. Positive and negative signals modulate formation of the Xenopus cement gland. Development. 1996;122: 2739–50. 8787748 10.1242/dev.122.9.2739

[11] Sive H, Bradley L. A sticky problem: the Xenopus cement gland as a paradigm for anteroposterior patterning. Dev Dyn. 1996;205: 265–80. 8850563 10.1002/(SICI)1097-0177(199603)205:3<265::AID-AJA7>3.0.CO;2-G

[12] Aberger F, Weidinger G, Grunz H, Richter K. Anterior specification of embryonic ectoderm: the role of the Xenopus cement gland-specific gene XAG-2. Mech Dev. 1998;72: 115–30. 9533957 10.1016/s0925-4773(98)00021-5

[13] Ramachandran V, Arumugam T, Wang H, Logsdon CD. Anterior gradient 2 is expressed and secreted during the development of pancreatic cancer and promotes cancer cell survival. Cancer Res. 2008;68: 7811–8. 10.1158/0008-5472.CAN-08-1320 18829536 PMC4429896

[14] Pohler E, Craig AL, Cotton J, Lawrie L, Dillon JF, Ross P, et al The Barrett’s antigen anterior gradient-2 silences the p53 transcriptional response to DNA damage. Mol Cell Proteomics. 2004;3: 534–47. 14967811 10.1074/mcp.M300089-MCP200

[15] Fritzsche FR, Dahl E, Dankof A, Burkhardt M, Pahl S, Petersen I, et al Expression of AGR2 in non small cell lung cancer. Histol Histopathol. 2007;22: 703–8. 17455144

[16] Zhang J-S, Gong A, Cheville JC, Smith DI, Young CYF. AGR2, an androgen-inducible secretory protein overexpressed in prostate cancer. Genes Chromosomes Cancer. 2005;43: 249–59. 15834940 10.1002/gcc.20188

[17] Park K, Chung YJ, So H, Kim K, Park J, Oh M, et al AGR2, a mucinous ovarian cancer marker, promotes cell proliferation and migration. Exp Mol Med. 2011;43: 91–100. 21200134 10.3858/emm.2011.43.2.011 PMC3047197

[18] Thompson DA, Weigel RJ. hAG-2, the human homologue of the Xenopus laevis cement gland gene XAG-2, is coexpressed with estrogen receptor in breast cancer cell lines. Biochem Biophys Res Commun. 1998;251: 111–6. 9790916 10.1006/bbrc.1998.9440

[19] Liu D, Rudland PS, Sibson DR, Platt-Higgins A, Barraclough R. Human homologue of cement gland protein, a novel metastasis inducer associated with breast carcinomas. Cancer Res. 2005;65: 3796–805. 15867376 10.1158/0008-5472.CAN-04-3823

[20] Fritzsche FR, Dahl E, Pahl S, Burkhardt M, Luo J, Mayordomo E, et al Prognostic relevance of AGR2 expression in breast cancer. Clin Cancer Res. 2006;12: 1728–34. 16551856 10.1158/1078-0432.CCR-05-2057

[21] Fletcher GC, Patel S, Tyson K, Adam PJ, Schenker M, Loader JA, et al hAG-2 and hAG-3, human homologues of genes involved in differentiation, are associated with oestrogen receptor-positive breast tumours and interact with metastasis gene C4.4a and dystroglycan. Br J Cancer. 2003;88: 579–85. 12592373 10.1038/sj.bjc.6600740 PMC2377166

[22] Edgell TA, Barraclough DL, Rajic A, Dhulia J, Lewis KJ, Armes JE, et al Increased plasma concentrations of anterior gradient 2 protein are positively associated with ovarian cancer. Clin Sci (Lond). 2010;118: 717–25. 10.1042/CS20090537 20136634

[23] Chung K, Nishiyama N, Yamano S, Komatsu H, Hanada S, Wei M, et al Serum AGR2 as an early diagnostic and postoperative prognostic biomarker of human lung adenocarcinoma. Cancer Biomark. 2012;10: 101–7. 10.3233/CBM-2012-0234 PMC13016256 22430137

[24] Kani K, Malihi PD, Jiang Y, Wang H, Wang Y, Ruderman DL, et al Anterior gradient 2 (AGR2): blood-based biomarker elevated in metastatic prostate cancer associated with the neuroendocrine phenotype. Prostate. 2013;73: 306–15. 10.1002/pros.22569 22911164

[25] Adam PJ, Boyd R, Tyson KL, Fletcher GC, Stamps A, Hudson L, et al Comprehensive proteomic analysis of breast cancer cell membranes reveals unique proteins with potential roles in clinical cancer. J Biol Chem. 2003;278: 6482–9. 12477722 10.1074/jbc.M210184200

[26] King ER, Tung CS, Tsang YTM, Zu Z, Lok GTM, Deavers MT, et al The anterior gradient homolog 3 (AGR3) gene is associated with differentiation and survival in ovarian cancer. Am J Surg Pathol. 2011;35: 904–12. 10.1097/PAS.0b013e318212ae22 21451362 PMC3095702

[27] Pascal LE, Vêncio RZN, Page LS, Liebeskind ES, Shadle CP, Troisch P, et al Gene expression relationship between prostate cancer cells of Gleason 3, 4 and normal epithelial cells as revealed by cell type-specific transcriptomes. BMC Cancer. 2009;9: 452 10.1186/1471-2407-9-452 20021671 PMC2809079

[28] Brychtova V, Zampachova V, Hrstka R, Fabian P, Novak J, Hermanova M, et al Differential expression of anterior gradient protein 3 in intrahepatic cholangiocarcinoma and hepatocellular carcinoma. Exp Mol Pathol. 2014;96: 375–81. 10.1016/j.yexmp.2014.04.002 24747240

[29] Gray TA, MacLaine NJ, Michie CO, Bouchalova P, Murray E, Howie J, et al Anterior Gradient-3: a novel biomarker for ovarian cancer that mediates cisplatin resistance in xenograft models. J Immunol Methods. 2012;378: 20–32. 10.1016/j.jim.2012.01.013 22361111

[30] Remmele W, Stegner HE. [Recommendation for uniform definition of an immunoreactive score (IRS) for immunohistochemical estrogen receptor detection (ER-ICA) in breast cancer tissue]. Pathologe. 1987;8: 138–40. 3303008

[31] Dahl E, Kristiansen G, Gottlob K, Klaman I, Ebner E, Hinzmann B, et al Molecular profiling of laser-microdissected matched tumor and normal breast tissue identifies karyopherin alpha2 as a potential novel prognostic marker in breast cancer. Clin Cancer Res. 2006;12: 3950–60. 16818692 10.1158/1078-0432.CCR-05-2090

[32] Elston EW, Ellis IO. Method for grading breast cancer. J Clin Pathol. 1993;46: 189–90. 8459046 10.1136/jcp.46.2.189-b PMC501162

[33] Waldmann A, Anzeneder T, Katalinic A. Patients and Methods of the PATH Biobank—A Resource for Breast Cancer Research. Geburtshilfe Frauenheilkd. 2014;74: 361–369. 25076793 10.1055/s-0033-1360263 PMC4078118

[34] The Cancer Genome Atlas Network. Comprehensive molecular portraits of human breast tumours. Nature. 2012;490: 61–70. 10.1038/nature11412 23000897 PMC3465532

[35] Cerami E, Gao J, Dogrusoz U, Gross BE, Sumer SO, Aksoy BA, et al The cBio cancer genomics portal: an open platform for exploring multidimensional cancer genomics data. Cancer Discov. American Association for Cancer Research; 2012;2: 401–4. 10.1158/2159-8290.CD-12-0095 22588877 PMC3956037

[36] Ten Haaf A, Bektas N, von Serenyi S, Losen I, Arweiler EC, Hartmann A, et al Expression of the glioma-associated oncogene homolog (GLI) 1 in human breast cancer is associated with unfavourable overall survival. BMC Cancer. 2009;9: 298 10.1186/1471-2407-9-298 19706168 PMC2753634

[37] Guiu S, Michiels S, André F, Cortes J, Denkert C, Di Leo A, et al Molecular subclasses of breast cancer: how do we define them? The IMPAKT 2012 Working Group Statement. Ann Oncol. 2012;23: 2997–3006. 10.1093/annonc/mds586 23166150

[38] Parker JS, Mullins M, Cheang MCU, Leung S, Voduc D, Vickery T, et al Supervised risk predictor of breast cancer based on intrinsic subtypes. J Clin Oncol. 2009;27: 1160–7. 10.1200/JCO.2008.18.1370 19204204 PMC2667820

[39] Makawita S, Dimitromanolakis A, Soosaipillai A, Soleas I, Chan A, Gallinger S, et al Validation of four candidate pancreatic cancer serological biomarkers that improve the performance of CA19.9. BMC Cancer. 2013;13: 404 10.1186/1471-2407-13-404 24007603 PMC3847832

[40] Innes HE, Liu D, Barraclough R, Davies MPA, O’Neill PA, Platt-Higgins A, et al Significance of the metastasis-inducing protein AGR2 for outcome in hormonally treated breast cancer patients. Br J Cancer. 2006;94: 1057–65. 16598187 10.1038/sj.bjc.6603065 PMC2361240

[41] Barraclough DL, Platt-Higgins A, de Silva Rudland S, Barraclough R, Winstanley J, West CR, et al The metastasis-associated anterior gradient 2 protein is correlated with poor survival of breast cancer patients. Am J Pathol. 2009;175: 1848–57. 10.2353/ajpath.2009.090246 19834055 PMC2774050

[42] Chevet E, Fessart D, Delom F, Mulot A, Vojtesek B, Hrstka R, et al Emerging roles for the pro-oncogenic anterior gradient-2 in cancer development. Oncogene. 2013;32: 2499–509. 10.1038/onc.2012.346 22945652

[43] Thuma F, Ngora H, Zöller M. The metastasis-associated molecule C4.4A promotes tissue invasion and anchorage independence by associating with the alpha6beta4 integrin. Mol Oncol. 2013;7: 917–28. 10.1016/j.molonc.2013.05.002 23727360 PMC5528461

[44] Ying L, Wang Q. Microfluidic chip-based technologies: emerging platforms for cancer diagnosis. BMC Biotechnol. 2013;13: 76 10.1186/1472-6750-13-76 24070124 PMC3849190

[45] Chignard N, Shang S, Wang H, Marrero J, Bréchot C, Hanash S, et al Cleavage of endoplasmic reticulum proteins in hepatocellular carcinoma: Detection of generated fragments in patient sera. Gastroenterology. 2006;130: 2010–22. 16762624 10.1053/j.gastro.2006.02.058

[46] Lee CH, Dershaw DD, Kopans D, Evans P, Monsees B, Monticciolo D, et al Breast cancer screening with imaging: recommendations from the Society of Breast Imaging and the ACR on the use of mammography, breast MRI, breast ultrasound, and other technologies for the detection of clinically occult breast cancer. J Am Coll Radiol. 2010;7: 18–27. 10.1016/j.jacr.2009.09.022 20129267

[47] Graham LJ, Shupe MP, Schneble EJ, Flynt FL, Clemenshaw MN, Kirkpatrick AD, et al Current Approaches and Challenges in Monitoring Treatment Responses in Breast Cancer. J Cancer. 2014;5: 58–68. 10.7150/jca.7047 24396498 PMC3881221

[48] Shupe MP, Graham LJ, Schneble EJ, Flynt FL, Clemenshaw MN, Kirkpatrick AD, et al Future Directions for Monitoring Treatment Responses in Breast Cancer. J Cancer. 2014;5: 69–78. 10.7150/jca.7048 24396499 PMC3881222

[49] Dershaw DD. Mammography in patients with breast cancer treated by breast conservation (lumpectomy with or without radiation). AJR Am J Roentgenol. 1995;164: 309–16. 7839960 10.2214/ajr.164.2.7839960

[50] Otieno BA, Krause CE, Latus A, Chikkaveeraiah B V, Faria RC, Rusling JF. On-line protein capture on magnetic beads for ultrasensitive microfluidic immunoassays of cancer biomarkers. Biosens Bioelectron. 2014;53: 268–74. 10.1016/j.bios.2013.09.054 24144557 PMC3855669

[51] Chikkaveeraiah B V, Bhirde AA, Morgan NY, Eden HS, Chen X. Electrochemical immunosensors for detection of cancer protein biomarkers. ACS Nano. 2012;6: 6546–61. 10.1021/nn3023969 22835068 PMC3429657

[52] Alix-Panabières C, Pantel K. Challenges in circulating tumour cell research. Nat Rev Cancer. 2014;14: 623–31. 10.1038/nrc3820 25154812

[53] Hüsemann Y, Geigl JB, Schubert F, Musiani P, Meyer M, Burghart E, et al Systemic spread is an early step in breast cancer. Cancer Cell. 2008;13: 58–68. 10.1016/j.ccr.2007.12.003 18167340

[54] Stott SL, Lee RJ, Nagrath S, Yu M, Miyamoto DT, Ulkus L, et al Isolation and characterization of circulating tumor cells from patients with localized and metastatic prostate cancer. Sci Transl Med. 2010;2: 25ra23. 10.1126/scitranslmed.3000403 PMC3141292 20424012

[55] Yu M, Bardia A, Wittner BS, Stott SL, Smas ME, Ting DT, et al Circulating breast tumor cells exhibit dynamic changes in epithelial and mesenchymal composition. Science. 2013;339: 580–4. 10.1126/science.1228522 23372014 PMC3760262

[56] Kloten V, Becker B, Winner K, Schrauder MG, Fasching PA, Anzeneder T, et al Promoter hypermethylation of the tumor-suppressor genes ITIH5, DKK3, and RASSF1A as novel biomarkers for blood-based breast cancer screening. Breast Cancer Res. 2013;15: R4 10.1186/bcr3375 23320751 PMC3672828

[57] Zhang P, Zou M, Wen X, Gu F, Li J, Liu G, et al Development of serum parameters panels for the early detection of pancreatic cancer. Int J Cancer. 2014;134: 2646–55. 10.1002/ijc.28584 24615168

[58] Sobin L, Wittekind C. TNM classification of malignant tumors 5th ed. New York: Wiley-Liss; 1997.

