# Supplementary material for: AGR3 in Breast Cancer: Prognostic Impact and Suitable Serum-Based Biomarker for Early Cancer Detection
Source: PLoS One. 2015 Apr 15;10(4):e0122106. doi: 10.1371/journal.pone.0122106 (PMC4398490; doi:10.1371/journal.pone.0122106)
Supplement: S1 Table — (DOC) [file pone.0122106.s001.doc]

| **S1 Table: Clinico-pathological parameters of 62 breast cancer specimens analysed in this study** | | | |
| --- | --- | --- | --- |
| **Parameter** | **Categorisation** | **n^a^ analysable** | **%** |
| Age at diagnosis: | median 63,5 years  (range 33-84) |  |  |
|  | <63,5 years | 31 | 50.0 |
|  | ≥63,5 years | 31 | 50.0 |
| Tumour size^b^ |  |  |  |
|  | pT1 | 35 | 56.5 |
|  | pT2 | 25 | 40.3 |
|  | pT3 | 2 | 3.2 |
|  | pT4 | 0 | 0 |
| Lymph node status^b^ |  |  |  |
|  | pN0 | 34 | 54.8 |
|  | pN1-3 | 27 | 43.6 |
|  | unknown | 1 | 1.6 |
| Histological tumour grade^c^ |  |  |  |
|  | G1 | 2 | 3.2 |
|  | G2 | 21 | 33.9 |
|  | G3 | 38 | 61.3 |
|  | unknown | 1 | 1.6 |
| Histological type |  |  |  |
|  | invasive ductal | 55 | 88.7 |
|  | invasive lobular | 5 | 8.1 |
|  | other | 2 | 3.2 |
| Oestrogen receptor status |  |  |  |
|  | negative (IRS^d^ 0-2) | 18 | 29.0 |
|  | positive (IRS^d^ 3-12) | 42 | 67.7 |
|  | unknown | 2 | 3.2 |
| Progesterone receptor status |  |  |  |
|  | negative (IRS^d^ 0-2) | 20 | 32.3 |
|  | positive (IRS^d^ 3-12) | 39 | 62.9 |
|  | unknown | 3 | 4.8 |
| HER2 status^e^ |  |  |  |
|  | negative | 53 | 85.5 |
|  | positive | 8 | 12.9 |
|  | unknown | 1 | 1.6 |
| ^a^Only female patients with primary, unilateral, invasive breast cancer were included. ^b^According to TNM classification by Sobin and Wittekind [58]. ^c^According to Bloom and Richardson, as modified by Elston and Ellis [32]. ^d^Immunoreactive score (IRS) according to Remmele and Stegner [30]. ^e^Overexpression of the *ERBB2* gene (Her-2/neu) was diagnosed analogously to the threshold of the DAKO-Score system based on IHC assay. Uncertain cases were additionally validated by FISH assay. Percentages may not sum-up to 100% due to rounding. | | | |
