# Supplementary material for: AGR3 in Breast Cancer: Prognostic Impact and Suitable Serum-Based Biomarker for Early Cancer Detection
Source: PLoS One. 2015 Apr 15;10(4):e0122106. doi: 10.1371/journal.pone.0122106 (PMC4398490; doi:10.1371/journal.pone.0122106)
Supplement: S2 Table — (DOC) [file pone.0122106.s002.doc]

| **S2 Table: Clinico-pathological parameters of 190 breast cancer specimens analysed in this study** | | | |
| --- | --- | --- | --- |
| **Parameter** | **Categorisation** | **n analysable** | **%** |
| Age at diagnosis: | median 58 years  (range 25-82) |  |  |
|  | <58 years | 92 | 48.4 |
|  | ≥58 years | 96 | 50.5 |
|  | unknown | 2 | 1.1 |
| Tumour size^a^ |  |  |  |
|  | pT1 | 61 | 32.1 |
|  | pT2 | 87 | 45.8 |
|  | pT3 | 12 | 6.3 |
|  | pT4 | 26 | 13.7 |
|  | unknown | 4 | 2.1 |
| Lymph node status^a^ |  |  |  |
|  | pN0 | 81 | 42.6 |
|  | pN1-3 | 102 | 53.7 |
|  | unknown | 7 | 3.7 |
| Histological tumour grade^b^ |  |  |  |
|  | G1 | 15 | 7.9 |
|  | G2 | 89 | 46.8 |
|  | G3 | 82 | 43.2 |
|  | unknown | 4 | 2.1 |
| Histological type |  |  |  |
|  | DCIS/IDC | 9 | 4.7 |
|  | invasive ductal | 173 | 91.1 |
|  | invasive lobular | 7 | 3.7 |
|  | other | 1 | 0.5 |
| Oestrogen receptor status |  |  |  |
|  | negative (IRS^c^ 0-2) | 44 | 23.2 |
|  | positive (IRS^c^ 3-12) | 101 | 53.2 |
|  | unknown | 45 | 23.7 |
| Progesterone receptor status |  |  |  |
|  | negative (IRS^c^ 0-2) | 107 | 56.3 |
|  | positive (IRS^c^ 3-12) | 51 | 26.8 |
|  | unknown | 32 | 16.9 |
| HER2 status^d^ |  |  |  |
|  | Negative (0; 1+; 2+) | 132 | 69.5 |
|  | Positive (3+) | 30 | 15.8 |
|  | unknown | 28 | 14.7 |
| ^a^According to TNM classification by Sobin and Wittekind [58]. ^b^According to Bloom and Richardson, as modified by Elston and Ellis [32]. ^c^Immunoreactive score (IRS) according to Remmele and Stegner [30]. ^d^Overexpression of the *ERBB2* gene (Her-2/neu) was diagnosed analogously to the threshold of the DAKO-Score system based on IHC assay. Percentages may not sum-up to 100% due to rounding. | | | |
