# Supplementary material for: AGR3 in Breast Cancer: Prognostic Impact and Suitable Serum-Based Biomarker for Early Cancer Detection
Source: PLoS One. 2015 Apr 15;10(4):e0122106. doi: 10.1371/journal.pone.0122106 (PMC4398490; doi:10.1371/journal.pone.0122106)
Supplement: S4 Table — (DOC) [file pone.0122106.s004.doc]

| **S4 Table: Clinico-pathological parameters of 40 breast cancer serum samples analysed for AGR2 protein concentration** | | | |
| --- | --- | --- | --- |
| **Parameter** | **Categorisation** | **n^a^ analysable** | **%** |
| Age at diagnosis: | median 60,5 years  (range 39-81) |  |  |
|  | <60,5 years | 20 | 50.0 |
|  | ≥60,5 years | 20 | 50.0 |
| Tumour size^b^ |  |  |  |
|  | pT1 | 23 | 57.5 |
|  | pT2 | 14 | 35.0 |
|  | pT3 | 3 | 7.5 |
| Lymph node status^b^ |  |  |  |
|  | pN0 | 31 | 77.5 |
|  | pN1-2 | 9 | 22.5 |
| Histological tumour grade^c^ |  |  |  |
|  | G1 | 3 | 7.5 |
|  | G2 | 33 | 82.5 |
|  | G3 | 3 | 7.5 |
|  | unknown | 1 | 2.5 |
| Histological type |  |  |  |
|  | invasive ductal | 32 | 80.0 |
|  | invasive lobular | 5 | 12.5 |
|  | IDC/ILC mixed | 2 | 5.0 |
|  | unknown | 1 | 2.5 |
| Oestrogen receptor status |  |  |  |
|  | negative (IRS^d^ 0-2) | 5 | 12.5 |
|  | positive (IRS^d^ 3-12) | 35 | 87.5 |
| Progesterone receptor status |  |  |  |
|  | negative (IRS^d^ 0-2) | 7 | 17.5 |
|  | positive (IRS^d^ 3-12) | 33 | 82.5 |
| HER2 status^e^ |  |  |  |
|  | negative (0; 1+; 2+) | 30 | 75.0 |
|  | positive (3+) | 10 | 25.0 |
| ^a^Only female patients with primary, unilateral, invasive breast cancer were included. ^b^According to TNM classification by Sobin and Wittekind [58]. ^c^According to Bloom and Richardson, as modified by Elston and Ellis [32]. ^d^Immunoreactive score (IRS) according to Remmele and Stegner [30]. ^e^Overexpression of the *ERBB2* gene (Her-2/neu) was diagnosed analogously to the threshold of the DAKO-Score system based on IHC assay. Percentages may not sum-up to 100% due to rounding. | | | |
