# Supplementary material for: AGR3 in Breast Cancer: Prognostic Impact and Suitable Serum-Based Biomarker for Early Cancer Detection
Source: PLoS One. 2015 Apr 15;10(4):e0122106. doi: 10.1371/journal.pone.0122106 (PMC4398490; doi:10.1371/journal.pone.0122106)
Supplement: S6 Table — (DOC) [file pone.0122106.s006.doc]

| **S6 Table: Primer sequences and PCR conditions for RNA expression analyses** | | |
| --- | --- | --- |
| **Primer** | **Sequence** | **Product size [bp]** |
|
| ***AGR3*  forward** | 5’-CTGGAGGATTGTCAATACTC-3’ | 103 |
| ***AGR3*  reverse** | 5’-GCATAAGGTTTAGCATGAT-3’ |  |
|  |  |  |
| ***GAPDH* forward** | 5’-GAAGGTGAAGGTCGGAGTCA-3’ | 289 |
| ***GAPDH* reverse** | 5’-TGGACTCCACGACGTACTCA-3’ |  |
|  | | |
| **Real-time PCR reaction volumes of 20 µl consisted of the following components:** | | |
| 5 µM forward primer, 5 µM reverse primer, 10 µl SYBR GRN Supermix and 1 µl of cDNA as PCR template. Cycle conditions: 95°C for 3 min, 40 cycles of 95°C for 30 s, 60°C for 20 s, 72°C for 30 s. bp: base pairs. | | |
|
|
