# Supplementary material for: AGR3 in Breast Cancer: Prognostic Impact and Suitable Serum-Based Biomarker for Early Cancer Detection
Source: PLoS One. 2015 Apr 15;10(4):e0122106. doi: 10.1371/journal.pone.0122106 (PMC4398490; doi:10.1371/journal.pone.0122106)
Supplement: S7 Table — (DOC) [file pone.0122106.s007.doc]

| **S7 Table: Clinico-pathological parameters in relation to *AGR3* mRNA expression^a^** | | | | |
| --- | --- | --- | --- | --- |
| **Parameter** | **n^b^** | ***AGR3* high** | ***AGR3* low** | **P-value^c^** |
| Total | 62 | 33 (53.2%) | 29 (46.8%) | - |
|  |  |  |  |  |
|  |  |  |  |  |
| Age at diagnosis |  |  |  |  |
| <63,5 years | 31 | 16 (51.6%) | 15 (48.4%) |  |
| ≥63,5 years | 31 | 17 (54.8%) | 14 (45.2%) | 1.000 |
| Tumour size^d^ |  |  |  |  |
| pT1 | 35 | 21 (60.0%) | 14 (40.0%) |  |
| pT2-3 | 27 | 12 (44.4%) | 15 (55.6%) | 0.306 |
| Lymph node status^d^ |  |  |  |  |
| pN0 | 34 | 16 (47.1%) | 18 (52.9%) |  |
| pN1-3 | 27 | 16 (59.3%) | 11 (40.7%) | 0.441 |
| Histological tumour grade^e^ |  |  |  |  |
| G1-2 | 23 | 18 (78.3%) | 5 (21.7%) |  |
| G3 | 38 | 14 (36.8%) | 24 (63.2%) | **0.003** |
| Histological type |  |  |  |  |
| invasive ductal | 55 | 28 (50.9%) | 27(49.1%) |  |
| invasive lobular | 5 | 4 (80.0%) | 1 (20.0%) | 0.359 |
| Oestrogen receptor status |  |  |  |  |
| negative (IRS^f^ 0-2) | 18 | 0 (0.0%) | 18 (100.0%) |  |
| positive (IRS^f^ 3-12) | 42 | 32 (76.2%) | 10 (23.8%) | **<0.001** |
| Progesterone receptor status |  |  |  |  |
| negative (IRS^f^ 0-2) | 20 | 2 (10.0%) | 18 (90.0%) |  |
| positive (IRS^f^ 3-12) | 39 | 30 (76.9%) | 9 (23.1%) | **<0.001** |
| HER2 status^g^ |  |  |  |  |
| negative | 53 | 31 (58.5%) | 22 (41.5%) |  |
| positive | 8 | 2 (25.0%) | 6 (75.0%) | 0.127 |
| ^a^*AGR3* high expression: ≥ 2 fold change in relation to median normal breast tissue expression. ^b^Only female patients with primary, unilateral, invasive breast cancer were included. ^c^Fisher’s exact test. ^d^According to TNM classification by Sobin and Wittekind [58]. ^e^According to Bloom and Richardson, as modified by Elston and Ellis [32]. ^f^Immunoreactive score (IRS) according to Remmele and Stegner [30]. Significant P-values are marked in bold face. ^g^Overexpression of the *ERBB2* gene (Her-2/neu) was diagnosed analogously to the threshold of the DAKO-Score system based on IHC assay. Uncertain cases were additionally validated by FISH assay. Percentages may not sum-up to 100% due to rounding. | | | | |
