# Supplementary material for: AGR3 in Breast Cancer: Prognostic Impact and Suitable Serum-Based Biomarker for Early Cancer Detection
Source: PLoS One. 2015 Apr 15;10(4):e0122106. doi: 10.1371/journal.pone.0122106 (PMC4398490; doi:10.1371/journal.pone.0122106)
Supplement: S8 Table — (DOC) [file pone.0122106.s008.doc]

| **S8 Table: Clinico-pathological parameters in relation to AGR3 protein expression^a^** | | | | |
| --- | --- | --- | --- | --- |
| **Parameter** | **n** | **AGR3 high** | **AGR3 low** | **P-value^b^** |
| Total | 190 | 101 (53.2%) | 89 (46.8%) | - |
|  |  |  |  |  |
|  |  |  |  |  |
| Age at diagnosis |  |  |  |  |
| <58 years | 92 | 44 (47.8%) | 48 (52.2%) |  |
| ≥58 years | 96 | 55 (57.3%) | 41 (42.7%) | 0.242 |
| Tumour size^c^ |  |  |  |  |
| pT1 | 61 | 32 (52.5%) | 29 (47.5%) |  |
| pT2-4 | 125 | 66 (52.8%) | 59 (47.2%) | 1.000 |
| Lymph node status^c^ |  |  |  |  |
| pN0 | 81 | 40 (49.4%) | 41 (50.6%) |  |
| pN1-3 | 102 | 55 (53.9%) | 47 (46.1%) | 0.555 |
| Histological tumour grade^d^ |  |  |  |  |
| G1-2 | 104 | 64 (61.5%) | 40 (38.5%) |  |
| G3 | 82 | 34 (41.5%) | 48 (58.5%) | **0.008** |
| Histological type |  |  |  |  |
| invasive ductal | 173 | 91 (52.6%) | 82 (47.4%) |  |
| invasive lobular | 7 | 6 (85.7%) | 1 (14.3%) | 0.126 |
| Oestrogen receptor status |  |  |  |  |
| negative (IRS^e^ 0-2) | 44 | 9 (20.5%) | 35 (79.5%) |  |
| positive (IRS^e^ 3-12) | 101 | 66 (65.3%) | 35 (34.7%) | **<0.001** |
| Progesterone receptor status |  |  |  |  |
| negative (IRS^e^ 0-2) | 107 | 51 (47.7%) | 56 (52.3%) |  |
| positive (IRS^e^ 3-12) | 51 | 33 (64.7%) | 18 (35.3%) | 0.060 |
| HER2 status^f^ |  |  |  |  |
| negative (0; 1+; 2+) | 132 | 74 (56.1%) | 58 (43.9%) |  |
| positive (3+) | 30 | 11 (36.7%) | 19 (63.3%) | 0.069 |
| ^a^Median immunoreactive score (IRS) according to Remmele and Stegner [30] was used as cut-off: AGR3 low (IRS 0-4), AGR3 high (IRS 6-12). ^b^Fisher’s exact test. ^c^According to TNM classification by Sobin and Wittekind [58]. ^d^According to Bloom and Richardson, as modified by Elston and Ellis [32]. ^e^Immunoreactive score (IRS) according to Remmele and Stegner [30]. Significant P-values are marked in bold face. ^f^Overexpression of the *ERBB2* gene (Her-2/neu) was diagnosed analogously to the threshold of the DAKO-Score system based on IHC assay. Percentages may not sum-up to 100% due to rounding. | | | | |
