# Supplementary material for: AGR3 in Breast Cancer: Prognostic Impact and Suitable Serum-Based Biomarker for Early Cancer Detection
Source: PLoS One. 2015 Apr 15;10(4):e0122106. doi: 10.1371/journal.pone.0122106 (PMC4398490; doi:10.1371/journal.pone.0122106)
Supplement: S1 Fig — (PPT) [file pone.0122106.s009.ppt]

## Slide 1
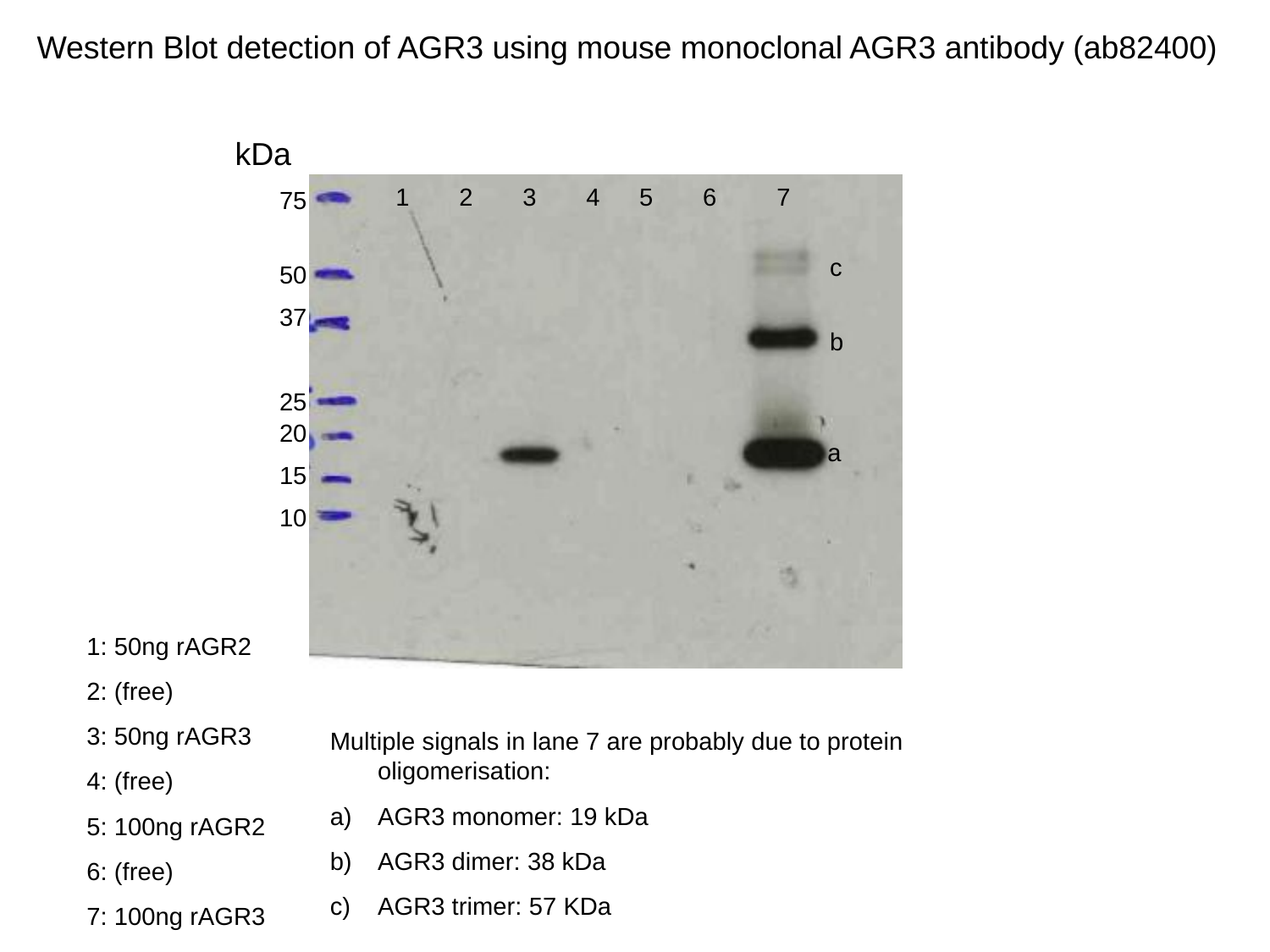

Western Blot detection of AGR3 using mouse monoclonal AGR3 antibody (ab82400)
kDa
1
2
3
4
5
6
7
75
c
50
37
b
25
20
a
15
10
1: 50ng rAGR2
2: (free)
3: 50ng rAGR3
4: (free)
5: 100ng rAGR2
6: (free)
7: 100ng rAGR3
Multiple signals in lane 7 are probably due to protein oligomerisation:
AGR3 monomer: 19 kDa
AGR3 dimer: 38 kDa
AGR3 trimer: 57 KDa
